# Supplementary material for: Silencing of TGFβ signalling in microglia results in impaired homeostasis
Source: Nat Commun. 2018 Oct 1;9:4011. doi: 10.1038/s41467-018-06224-y (PMC6167353; doi:10.1038/s41467-018-06224-y)
Supplement: Supplementary file 1 — Supplementary Information [file 41467_2018_6224_MOESM1_ESM.pdf]

Supplementary material

Silencing of TGFβ signalling in microglia results in impaired homeostasis

Zöller et al.

Supplementary Figure 1

Experimental design and validation of genomic recombination

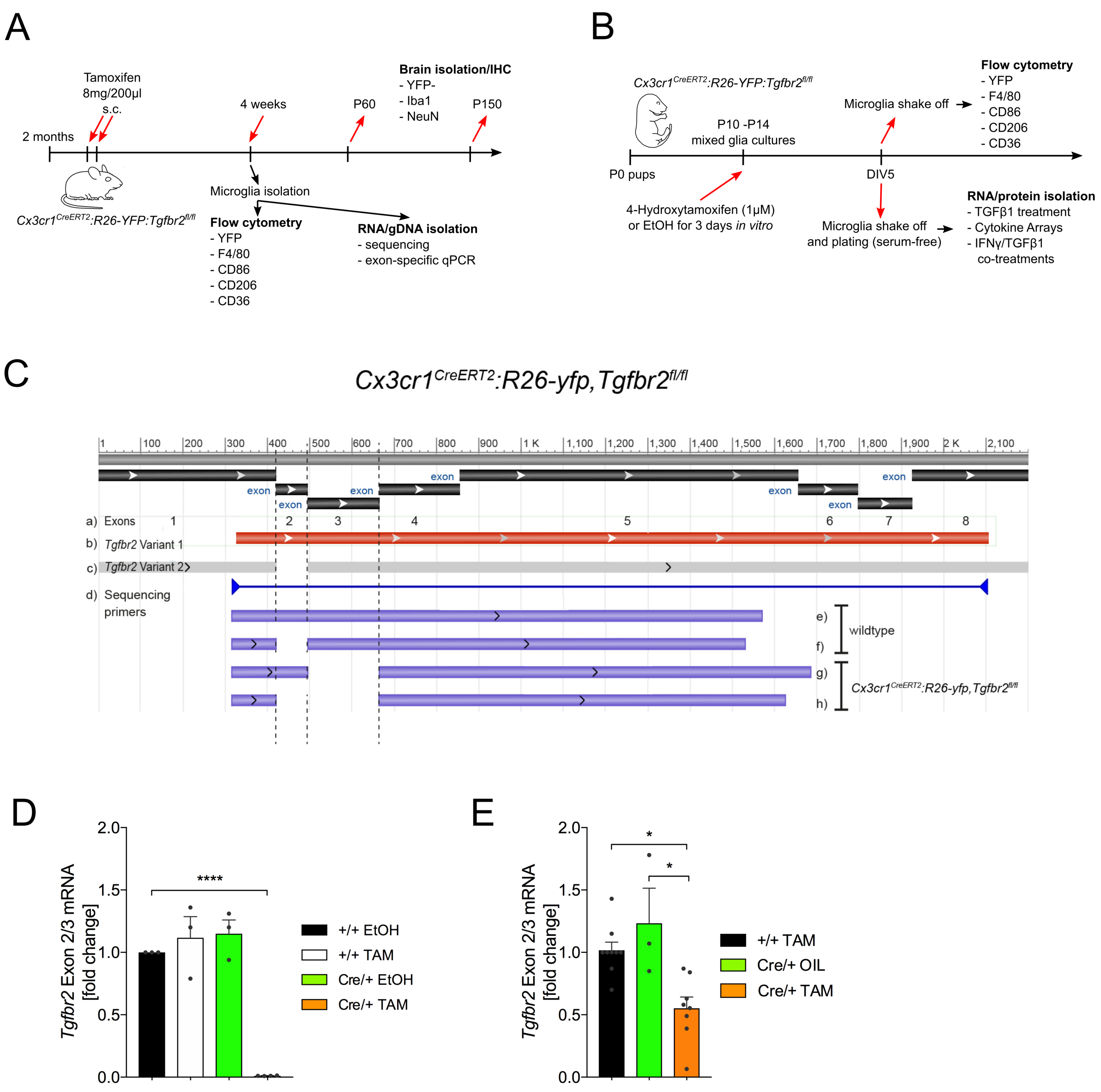

(**A**) Timeline displaying time points and concentrations of tamoxifen injections and experimental approaches for *in vivo*. (**B**) Timeline for *in vitro* recombinations and experimental designs. (C) NCBI nucleotide blast of sequencing data from wild type and *Cx3cr1*^{CreERT2}*:R26-yfp,Tgfbr2*$^{fl/fl}$ microglia. Exons of the *Tgfbr2* gene are depicted in black, *Tgfbr2* transcript is depicted in red. *Tgfbr2* transcript variants with (Variant 1) and without Exon 2 (Variant 2) in wildtype microglia and sequences of *Tgfbr2* variants after TAM-induced recombination. (**D**) Tgfbr2 Exon2/3 expression after TAM-induced recombination *in vitro*. Data are given as means ± SEM (+/+ EtOH n=3, +/+ TAM n=2, Cre/+ EtOH n=2, Cre/+ TAM n=4). P-value derived from one-way ANOVA is ****$p<0.0001$. (**E**) Tgfbr2 Exon2/3 expression after TAM-induced recombination *in vivo*. Data are given as means ± SEM (+/+ TAM n=5, Cre/+ TAM n=5). P-value derived from one-sample t-tests is *$p<0.05$.

Supplementary Figure 2

Validation of microglia recombination *in vivo*

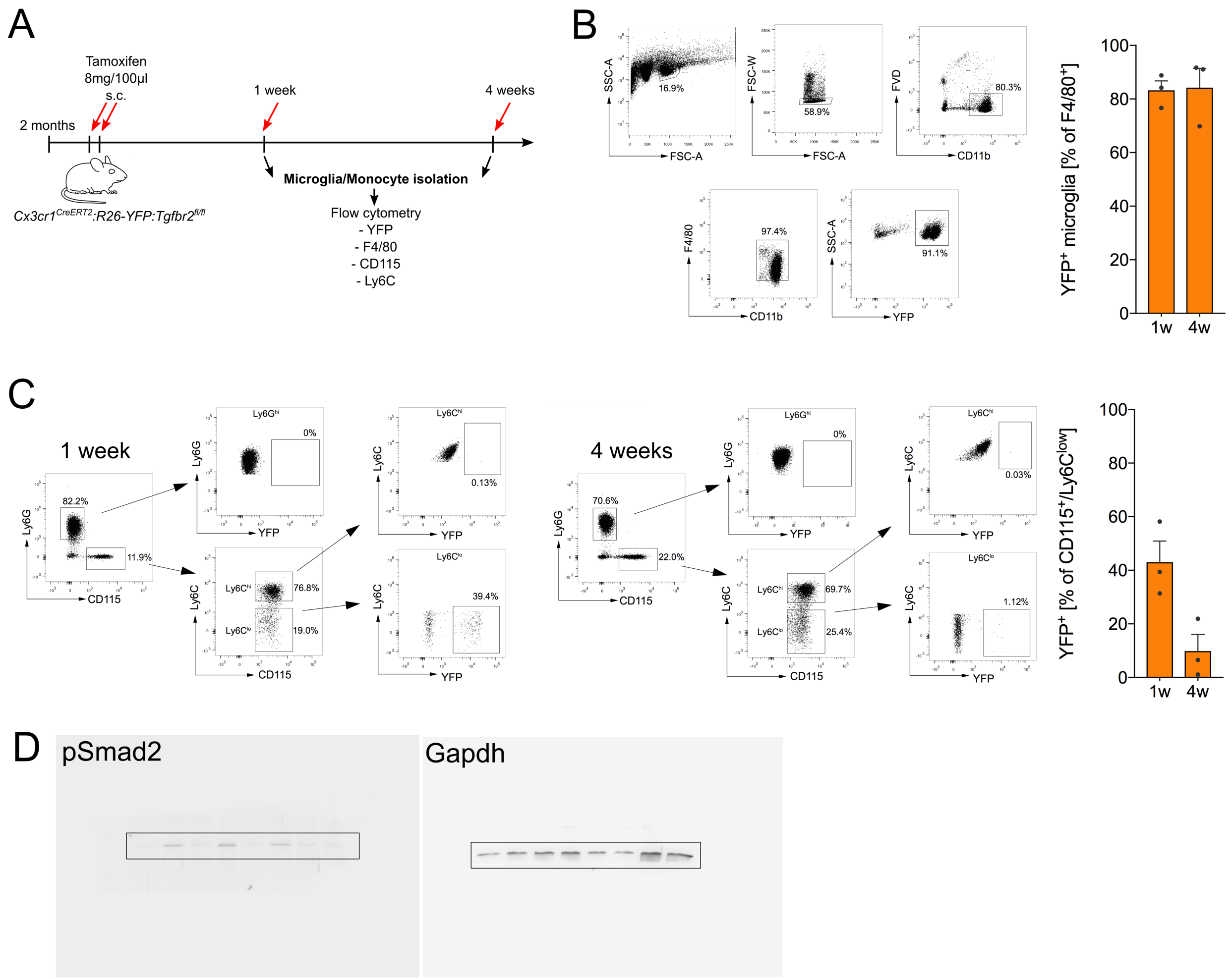

(A) A timeline showing the experimental design to analyse recombination in microglia and blood monocytes in *Cx3cr1^{CreERT2}:R26-yfp,Tgfbr2$^{fl/fl}$* mice. (**B**) Gating strategy and detection of YFP$^+$ microglia to monitor recombination efficacies 1 week (1w) and 4 weeks (4w) after TAM injections. (**C**) Gating strategy and detection of YFP$^+$ monocytes 1 week and 4 weeks after TAM-induced recombination. Data are given as means ± SEM (Cre/+ TAM n=3 per time point).

Supplementary Figure 3

Analysis of microglia morphology

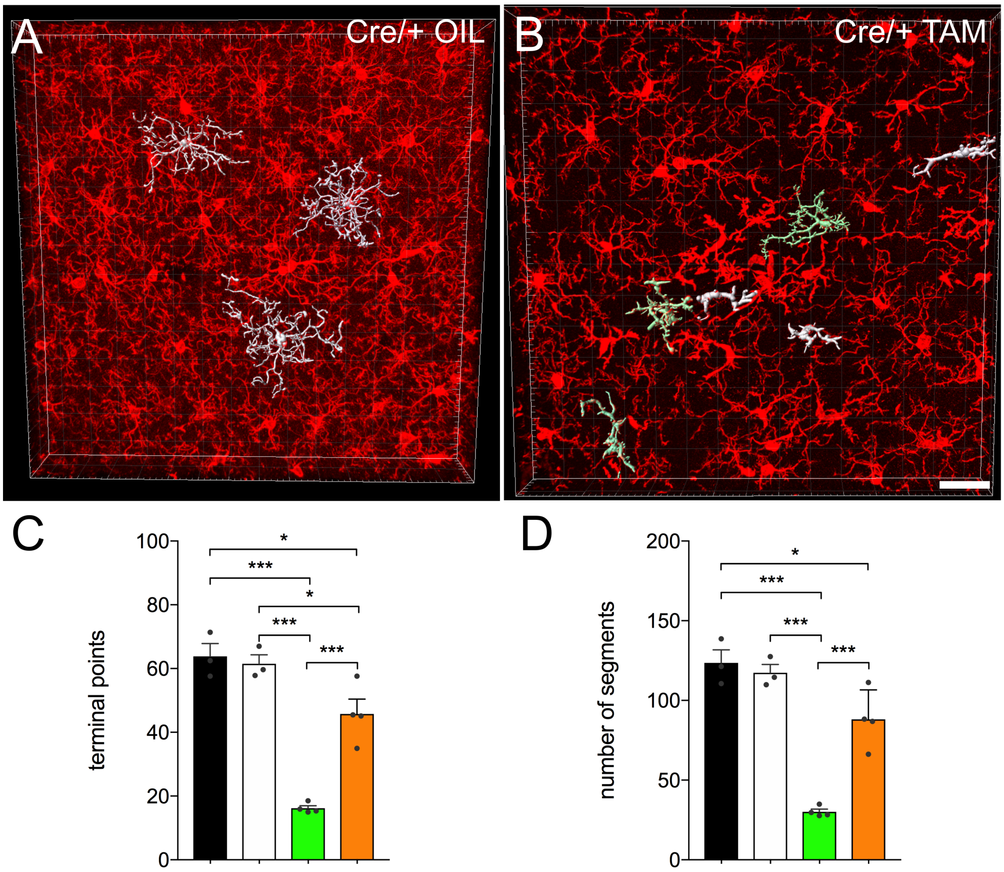

Microglia morphology after Imaris-based threedimensional reconstruction in z-stacks from Cre/+ OIL (**A**) and Cre/+ TAM (**B**) cortical microglia. Distinct morphological changes of Iba1^{+} microglia 4 weeks after TAM-induced deletion of *Tgfbr2* in Cre/+ TAM knockout mice were subdivided into „Bushy“ and „Hypertrophied“. Imaris-based automated quantification of microglial terminal points (**B**) and numbers of segments (**C**). Data are presented as means ± SEM of 20 cells in 3-4 animals per group. P-values derived from one-way ANOVA are *p<0.05, and ***p<0.001.

Supplementary Figure 4

TGFβ1-regulated expression of microglia surface receptors *in vitro*

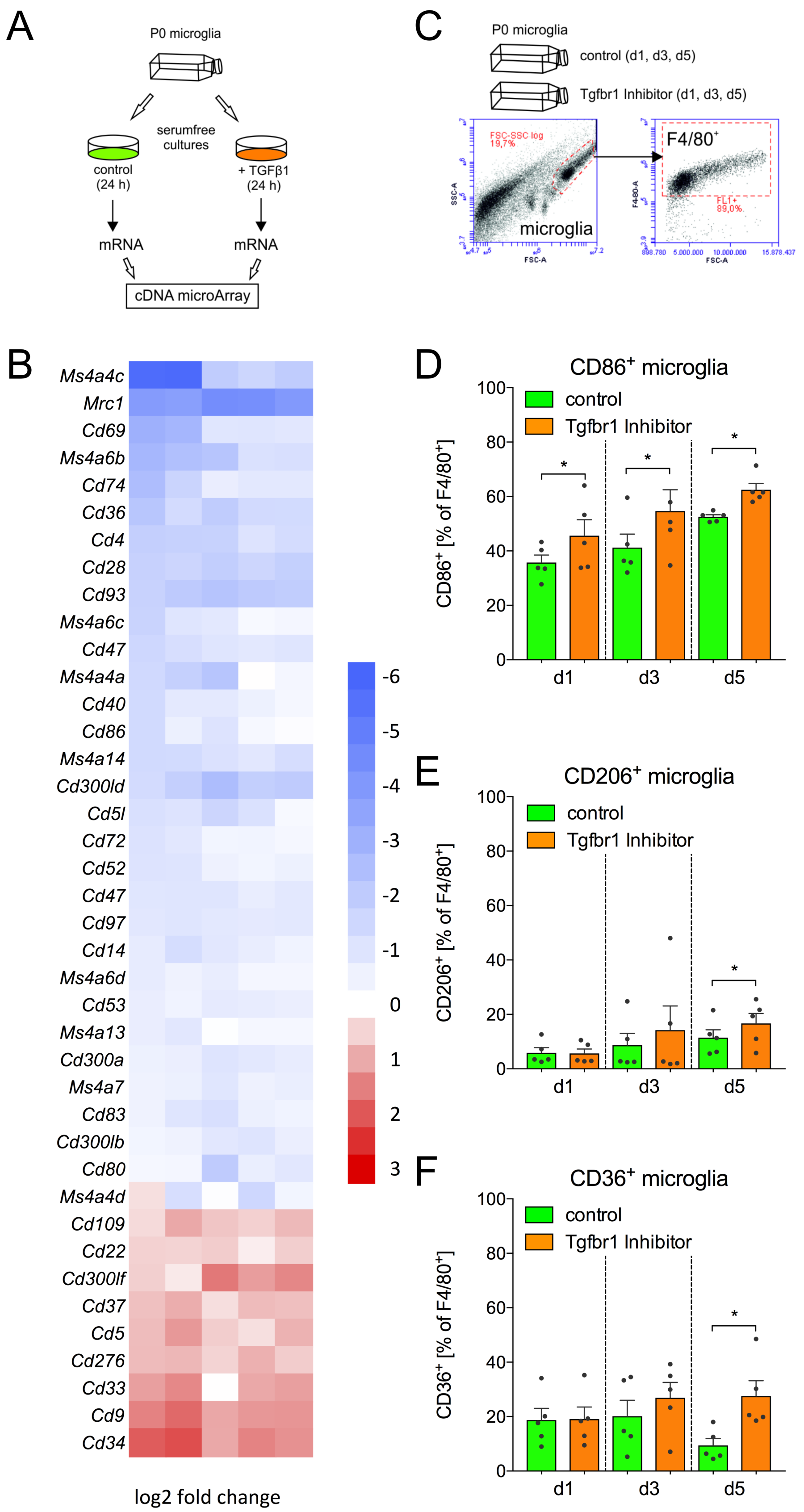

(**A**) Scheme illustrating the experimental strategy to obtain mRNA from control (serumfree culture medium, 24 h) and TGFβ1-treated (5 ng/ml, 24 h) primary microglia for cDNA microArrays. (**B**) Heatmap analysis showing log2 transformed fold changes of TGFβ1-regulated genes coding for membrane-localized proteins. Data are presented from 5 independent *in vitro* experiments. Fold changes with p<0.05 derived from paired Bayes T-Test were included in the heatmap. (**C**) Validation of TGFβ1-regulated genes after inhibition of TGFβ signalling. mixed glia cultures were treated with Tgfbr1 inhibitor (500 mM) for 1 day (d1), 3 days (3d), and 5 days (5d). Microglia were isolated (shaking method) and immediatelly stained against F4/80 for flow cytometry analysis. Percentages of CD86$^+$ (**D**), CD206$^+$ (**E**), and CD36$^+$ (**F**) microglia at different time points after inhibition of TGFβ signalling. Data are given as means ± SEM from five independent experiments for each time point. P-values derived from one-sample t-tests are *p<0.05.

Supplementary Figure 5

TGFβ1-regulated expression of cytokines/chemokines and microglia-specific genes

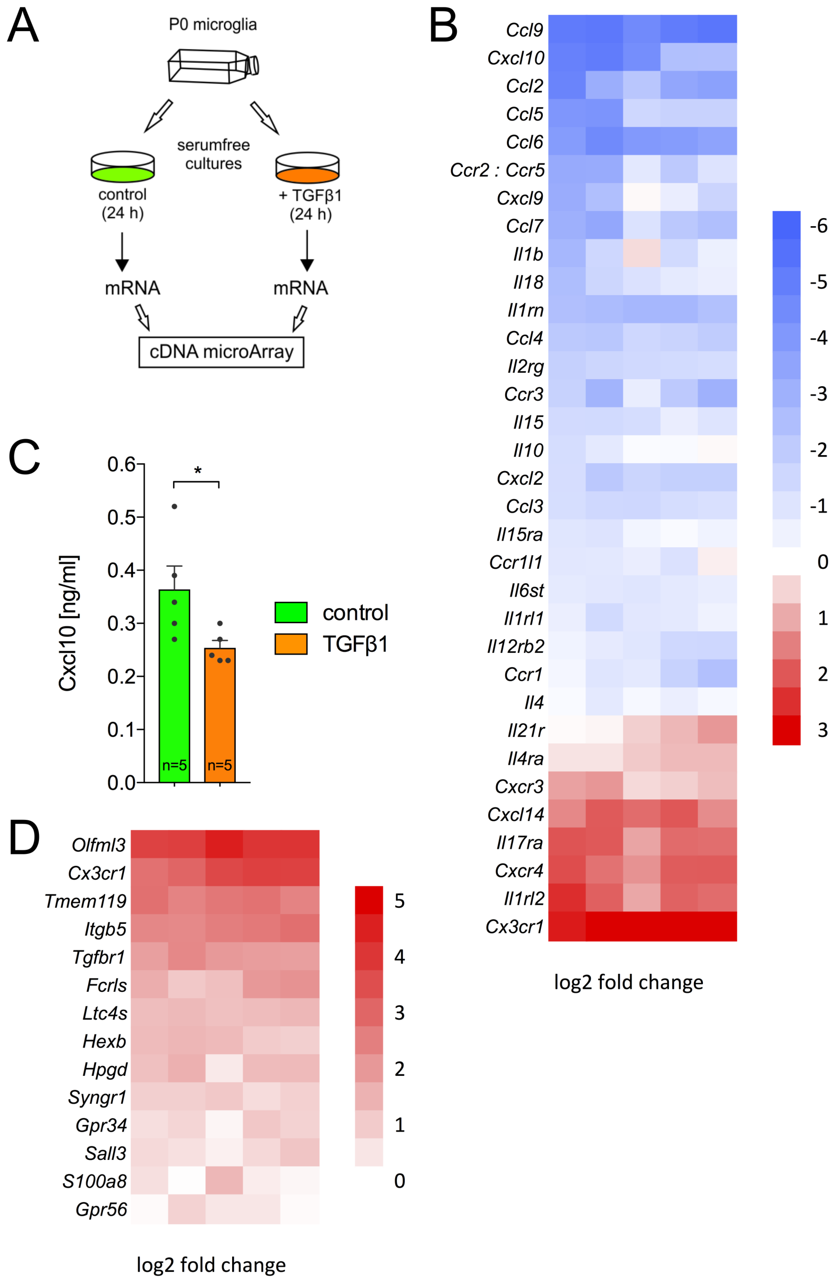

(**A**) Scheme illustrating the experimental strategy to obtain mRNA from control (serumfree culture medium, 24 h) and TGFβ1-treated (5 ng/ml, 24 h) primary microglia for cDNA microArrays. (**B**) Heatmap analysis showing log2 transformed fold changes of TGFβ1-regulated genes coding for cytokines, chemokines and respective receptors. Data are presented from 5 independent *in vitro* experiments. Fold changes with $p<0.05$ derived from paired Bayes t-test were included in the heatmap. (**C**) ELISA demonstrating reduced Cxcl10 release from TGFβ1-treated (5 ng/ml, 24 h) primary microglia. Data are given as means ± SEM from five independent experiments. P-value derived from one-sample t-tests is $*p<0.05$. (**D**) Heatmap illustrating TGFβ1-regulated microglia-specific genes. Data are presented from as log2 fold changes from 5 independent experiments. Fold changes with p-values <0.05 derived from Bayes t-test were included in the depicted heatmap.

Supplementary methods

Microarray analysis

Integrity of RNA was analysed by capillary electrophoresis using a 2100 Bioanalyzer (Agilent Technologies, CA). Samples with an RNA integrity number (RIN) between 8.6 and 10 (mean 9.45) were further processed using the Affymetrix GeneChip Whole Transcript Sense Target Labeling Assay as described by the manufacturer. 500 ng of total RNA were reverse transcribed with (N6)-random primers coupled to a T7 RNA Polymerase promotor sequence. After second-strand synthesis, double-stranded cDNAs were amplified overnight by *in-vitro*-transcription using T7 RNA Polymerase. cRNA was purified and used as template for a second cycle of reverse transcription with (N6) random primers. dUTP was incorporated into the cDNA, and after hydrolysis of the cRNA with RNAse H, cDNA was purified and fragmented using Uracil-DNA Glycosylase (UDG) and human apurinic/apyrimidinic Endonuclease 1 (APE1). cDNA fragments were labeled at their 3' end with biotin using Terminal Deoxynucleotidyl Transferase (TdT). Labeled fragments were hybridised to the arrays for 16h at 45°C with 60 rpm in an Affymetrix Hybridization oven 640. The arrays were scanned after washing and staining using the Affymetrix GeneChip Scanner 3000 7G. CEL-files were produced from the raw data with Affymetrix GeneChip Command Console Software Version 3.0. Genedata Expressionist software was further used for data analysis. CEL files were imported into the Refiner (Version 6.1) module of Expressionist where GC background subtraction was performed using antigenomic background probes. Quantile normalisation and probe summarisation was performed with the Bioconductor RMA condensing algorithm as implemented in Refiner[1]. Differentially expressed genes between the groups were identifed and the paired Bayes T-test (CyberT)[2] with the Bayes Confidence Estimate Value set to 10 and a window size of 101 genes as well as 100 % valid values in each group was used (Analyst module, version 2.2.6b). Benjamini-Hochberg q-value was calculated[3] in order to control the false discovery rate. We then used the "2 groups paired" activity of Analyst to calculate

the paired Effect size score between the experimental groups. Only genes from the categories "main" and "unmapped" (see Affymetrix transcript annotation NA32) were included in the analysis.

Microglia and blood monocyte isolation

Microglia were isolated using the Percoll gradient method. Blood monocytes were collected from 1 ml blood which was obtained from the left ventricle and transferred into a 2.0 ml tube containing 0.5M EDTA at 4°C. After centrifugation, the supernatant was discarded and 1mL 1X RBC Lysis buffer (00-4333-57, eBioscience) was added, followed by an incubation at 4°C for 10 min. Then cells were washed with PBS containing 2 % FCS and 10 mM EDTA before staining. Cells were stained with: FC receptor blocking antibody (clone 2.4G2, 553142, BD), FVD: eFluor 506 (65-0866-14, eBioscience), F4/80: PE(clone: BM8, 12-4801-82, eBioscience), CD45: APC-eFluor 780 (clone: 30-F11, 47-0451-82, eBioscience), CD11b: BV421(clone:M1/70, 101236, BioLegend), Ly6C: PerCP-Cy5.5 (clone:AL-21, 560525, BD Bioscience), Ly6G: PE (clone:1A8, 551461, BD Bioscience), CD115: PE-Cy7(clone: AFS98, 25-1152-82, eBioscience) at a dilution of 1:200 at 4°C for 30 min. After final washing, cells were analyzed using FACS Canto II (BD Biosciences). Viable cells were gated by staining with Fixable Viability Dye (eBioscience).

ELISA

Levels of Cxcl10 in the medium of primary microglia were detected using an ELISA Development Kit (Peprotech, Hamburg, Germany) according to the manufacturer´s instructions. Colour reaction was performed using 2,2'-azino-bis(3-ethylbenzothiazoline-6-sulphonic acid) substrate (ABTS, Sigma-Aldrich, Germany) for 30 minutes in the dark. Absorbance was detected using a Multiskan FC plate reader (Thermo Fischer) at the absorption of 405 nm. Concentrations of Cxcl10 were calculated from the standard curve using the GraphPad Prism6 software (GraphPad Software Inc.).

Sequencing

cDNA of primary microglia was amplified with primers *Tgfbr2Seq* forward 5′-TTAACTCGAGGGCTGCCATGGGTCG-3′, *Tgfbr2Seq* reverse 5′-GACGTCGACATTTTGGTAGTGTTCAGCA-3′. Purified PCR products were shipped for sequencing at GATC Biotech AG “LightRun” (www.gatc-biotech.com). Obtained sequences were analysed using SnapGene and NCBI BioSystems database (www.ncbi.nlm.nih.gov).

Supplementary references

1. Irizarry, R. A. et al. Exploration, normalization, and summaries of high density oligonucleotide array probe level data. *Biostatistics* **4**, 249–264 (2003).
2. Baldi, P. & Long, A. D. A Bayesian framework for the analysis of microarray expression data: regularized t -test and statistical inferences of gene changes. *Bioinformatics* **17**, 509–519 (2001).
3. Benjamini, Y., Hochberg. Y. Controlling the false discovery rate: a practical and powerful approach to multiple testing. *J Royal Stat Soc B* **57**: 289 (1995).
